# Synchronous Bilateral Breast Cancer: A Case Report Piloting and Evaluating the Implementation of the AI-Powered Large Language Model (LLM) ChatGPT

**DOI:** 10.7759/cureus.37587

**Published:** 2023-04-14

**Authors:** Himani R Naik, Andrew D Prather, Grzegorz T Gurda

**Affiliations:** 1 Gundersen Medical Foundation, Gundersen Lutheran Medical Center, La Crosse, USA; 2 Radiology, Gundersen Lutheran Medical Center, La Crosse, USA; 3 Biology, University of Wisconsin-La Crosse, La Crosse, USA; 4 Pathology, Gundersen Lutheran Medical Center, La Crosse, USA

**Keywords:** chatgpt aided case report, chatgpt, breast cancer risk, multifocal, bilateral, ductal carcinoma, breast cancer

## Abstract

Primary breast carcinoma is the most common cancer type in women, and although bilateral synchronous breast cancers (s-BBC) remain quite rare, the reported incidence may increase with the adoption of more sensitive imaging modalities. Here, we present a case of histomorphological and clinically distinct s-BBC, together with a discussion of clinical management decisions, prognosis, and treatment standards and how these relate to outcomes vis-à-vis more established standards in unifocal breast carcinoma. The case report also constitutes a pilot and formal evaluation of a large language model (LLM) of ChatGPT as a tool to aid in generating a single patient case report.

## Introduction

The text below was generated through a combination of ChatGPT queries and manual (human) writing and editing. The full constituent components of the text, including analysis of the writing, are presented in the appendices. 

Synchronous bilateral breast cancer (s-BBC) is a rare presentation of breast cancer, accounting for 1%-3% of all breast cancer cases. s-BBC is typically defined as the presence of invasive or in situ breast cancer in both breasts diagnosed within six months of each other [1,2]. The diagnosis of s-BBC can be challenging, as the tumors can present at different stages and have different clinical presentations and histopathological characteristics. The prognosis and treatment options for s-BBC depend on several factors, including the stage, histological grade, and other prognostic factors of each individual tumor. Here, we present a case report of a patient with s-BBC, one intermediate-risk ductal carcinoma in the right breast with positive right axillary lymph nodes, and one low-risk ductal carcinoma with lobular features in the left breast with negative left axillary lymph nodes. We discuss the clinical management, prognosis, and treatment implications of s-BBC.

## Case presentation

**Figure 1:**
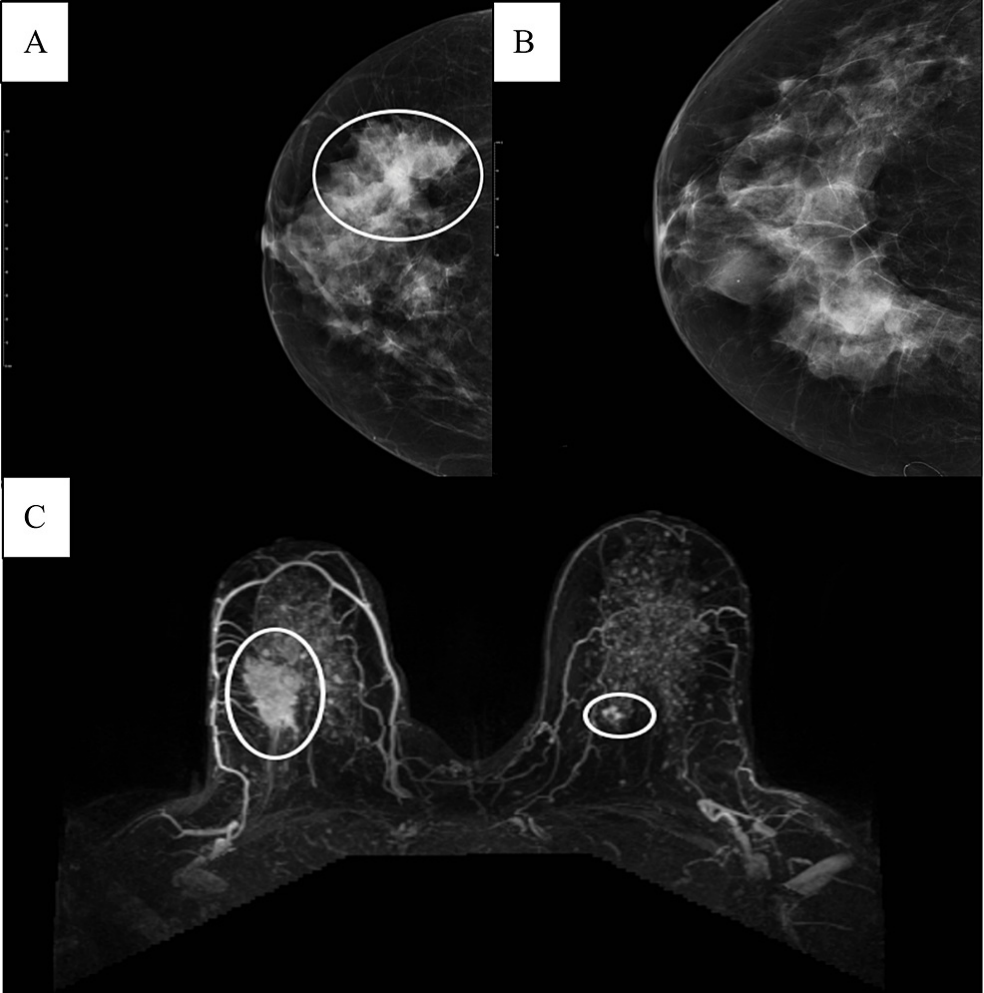
Pre-operative imaging A. Mammography, right breast, cranio-caudal (CC) view, white oval to indicate lesion area B. Mammography, right breast, cranio-caudal (CC) view, no definite findings C. Magnetic resonance imaging (MRI), post-contrast T1, white ovals to indicate lesions bilaterally.

A 58-year-old woman presented with a history of a painless lump in her right breast. Mammography and ultrasound showed a 4.5-cm suspicious mass in the mid-posterior upper outer quadrant of the right breast (Figure 1A) and initially no significant suspicious lesions in the left breast (Figure 1B). Following a malignant diagnosis of the right breast mass upon ultrasound (US)-guided needle core biopsy, a contrast-enhanced diagnostic magnetic resonance imaging (MRI) of both breasts was performed and identified an additional 1.5 cm of enhancing lesion in the upper inner quadrant of the left breast (Figure 1C). 

**Figure 2:**
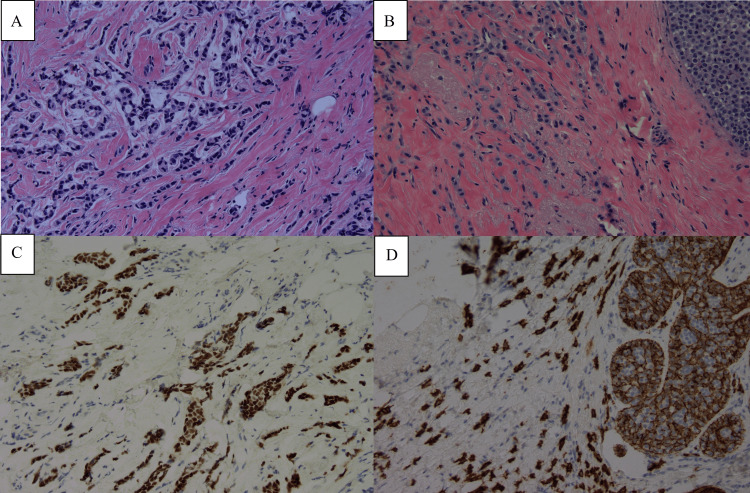
Pre-operative pathology (needle core biopsies, right and left breast) A. H&E, 20X, histomorphology of right-sided ductal carcinoma B. Estrogen receptor (ER) strong positivity, progesterone receptor (PR) weak positivity (not shown) C. H&E, 20X, histomorphology of right-sided ductal carcinoma with lobular features and adjacent atypical lobular hyperplasia (ALH) D. Partial loss of E-cadherin membranous staining within ALH, preserved within invasive carcinoma; estrogen receptor (ER) strong positivity; progesterone receptor (PR) weak-moderate positivity (not shown)

An MRI-guided needle biopsy was performed, establishing the diagnosis of invasive ductal carcinoma with lobular carcinoma of the left breast. The tumors were both estrogen receptor (ER) strongly positive, progesterone receptor (PR) weakly to moderately positive, and HER2/neu-negative (Figure 2); an E-cadherin immunohistochemical stain showed strong, preserved membranous staining, supporting the histomorphologic impression of a predominantly ductal phenotype but also focal lobular neoplasia in situ (ALH) of the left breast. 

The patient had no family history of breast or ovarian cancer, and her risk assessment (the Gail model) indicated a low risk. The patient also met with a genetic counselor and underwent germline testing for high-prevalence breast cancer risk genes (Invitae, San Francisco, CA), but no pathologic variants (category 1 or 2) were found. The patient was diagnosed with s-BBC and underwent a multidisciplinary evaluation at our institutional breast cancer tumor board to determine the best treatment plan.

The patient underwent a bilateral skin-sparing mastectomy with reconstruction, bilateral sentinel lymph node biopsy, and axillary lymph node dissection. The final pathology revealed a 4.5 cm intermediate-grade (Nottingham combined grade 2 of 3) ductal carcinoma with 7 of 21 positive right axillary lymph nodes in the right breast and a 1.4 cm low-grade (Nottingham combined grade 1 of 3) ductal carcinoma with lobular features in the left breast with negative left axillary lymph nodes. The patient’s pathologic staging (AJCC 8th ed.) was, therefore, pT2 N2a on the right side and pT1c pN0(sn) on the left side.

The patient received adjuvant radiation therapy to the right breast and axilla, as well as systemic adjuvant AC-T chemotherapy, i.e., doxorubicin hydrochloride (Adriamycin) and cyclophosphamide, followed by paclitaxel (Taxol) and subsequent adjuvant endocrine therapy with a non-steroidal aromatase inhibitor (AI), anastrozole. The right breast was closely monitored with regular radiographic screening and clinical examinations of the reconstructed breast tissue and implant, and there has been no evidence of disease recurrence during the five-year follow-up period.

## Discussion

The management of s-BBC depends on several factors, including the stage of the disease, the histopathological characteristics of the tumors, the patient's health status, including age and comorbidities, as well as the patient's own health management preferences. Treatment options include breast-conserving surgery, mastectomy, radiation therapy, and systemic therapy with chemotherapy, endocrine therapy, or targeted therapy.

Breast-conserving surgery (BCS) is a reasonable option for patients with s-BBC, provided that the tumors can be completely excised with negative margins. The prognosis of s-BBC has long been considered to be generally worse than that of unilateral breast cancer, with worse overall survival than either unilateral or metachronous breast cancer likely due to an earlier and perhaps higher risk of distant metastasis [1]. This has been re-appraised in more recent studies with larger sample sizes, stringent data collection at the outset, longer follow-up, and multivariate adjustment for tumor biology to show that the inferior prognosis of s-BBC may not be due to the higher aggressiveness of cancers in this setting per se, but the combined detrimental effect due to simultaneous malignancies [2,3]. The presence of positive lymph nodes is associated with a worse prognosis, and patients with positive lymph nodes should receive adjuvant chemotherapy in addition to surgery and radiation therapy [4]. In this case, the patient received adjuvant chemotherapy based on the intermediate risk of right breast cancer.

Mastectomy may be considered or recommended in patients with large or multifocal tumors, an extensive intraductal component, or a strong family history of breast cancer [4]. Radiation therapy is recommended for s-BBC patients who undergo breast-sparing surgery such as lumpectomy to reduce the risk of local recurrence. Bilateral breast irradiation can be challenging with different fields and dose requirements for variably aggressive tumors at two sites, but a hypofractionated schedule is technically feasible, can minimize acute toxicity/side effects, and shows no increase in the risk of significant late effects (for instance, secondary malignancies such as angiosarcomas), though the study conclusions should be viewed with caution due to the relatively short follow-up [5]. 

Systemic therapy with chemotherapy, endocrine therapy, or targeted therapy may be considered based on the histological characteristics of the tumors and the patient's overall health. In our case, the patient received adjuvant endocrine therapy with an AI (anastrozole) given the hormone receptor-positive status of both tumors, particularly the larger, more biologically aggressive right-sided tumor. In a retrospective study of 1,214 patients with s-BBC, there was a similar range of distribution for tumor size, pathological grade, ER positivity, and axillary lymph node involvement, and when corrected with multivariate competing risk models using both tumor sites as opposed to unilateral breast cancer, there was no difference in overall survival (Risk Ratio (RR) = 1.01, CI: 1.08-1.57, p=0.93) [2]. Similarly, in a large multi-site clinical trial based on the utilization of a 70-gene signature in low-risk breast cancer patients, 238 s-BBC patients with the multifocal disease showed a slightly elevated genomic risk profile versus unifocal breast cancer patients (high risk of 22.7% vs. 17.3%, odds ratio [OD] = 1.45, p=0.038), but no association in disease-free survival (DFS 96.9% vs. 97.1%, hazard ratio [HR] = 1.55, p=0.172) [3], albeit with some limitations as to the general applicability of the study due to the characteristics of the patient accrual. Nearly all studies seem to agree with the generally accepted paradigm that invasive lobular carcinoma and lobular neoplasia in situ (ALH/LCIS) are surrogate risk markers for the development of future breast cancer (in both breasts) and that lobular carcinoma in situ (LCIS) is a non-obligate precursor of breast cancer. In the studies discussed thus far, the lobular phenotype was enriched in s-BBC, representing 11% in the unilateral setting versus 13.7%-15.6% in s-BBC [2] or 6.6% in metachronous BBC versus 8.6% in s-BBC [1]. For the patient presented in this case report, there was documented lobular neoplasia in situ (ALH) in the smaller tumor, as well as lobular features histomorphologically, albeit this characterization is difficult to stringently and uniformly apply. In terms of late recurrence and metastasis, there also does not appear to be a significant difference in the anatomic site and distribution of late/distal metastasis versus metachronous breast cancer (in descending order, bone, lymph nodes, lungs, liver, and brain, among others) [1]. Lastly, in the setting of s-BBC, consideration of metastasis to the contralateral breast rather than two synchronous primary cancers should be considered, especially if the tumors appear histomorphological similar, have similar receptor status, are of high grade/stage, have axillary metastasis on one side, but lack carcinoma in situ (DCIS/LCIS) in the contralateral breast. More rigorous analytical approaches, such as next-generation sequencing, have shown that about 5% of s-BBC may indeed constitute metastatic disease involving the contralateral breast rather than multifocal synchronous cancers [6]. Although these advanced molecular diagnostics approaches are informative in terms of further prognosis and management, they can be difficult to execute in a typical clinical setting and, due to some limitations, may still give equivocal results.

As stated in the case presentation, the patient underwent genetic counseling and underwent broad-panel germline testing with negative results. Per current national comprehensive cancer network (NCCN) guidelines (version 3.2023), an indication for testing for high penetrance cancer susceptibility genes (BRCA1/2, CDH1, PALB2, PTEN, and TP53) is indicated in women with multiple primary breast cancers, regardless of age, and this includes both s-BBC and metachronous breast cancer [4].

There is limited evidence on the optimal follow-up strategy for patients with s-BBC. The general approach is to follow the patient clinically after surgery, with imaging at least once or twice within the next two years [7]. Magnetic resonance imaging (MRI) may be considered for high-risk patients, such as those with a strong family history of breast or ovarian cancer or with dense breast tissue [8]. Adoption of more advanced and sensitive imaging techniques such as MRI may lead to increased detection of what would otherwise be clinically and grossly (by surgical pathology) occult carcinomas and thus lead to an increased reported incidence of s-BBC in the future.

## Conclusions

s-BBC is a rare presentation of breast cancer that requires a careful diagnostic and clinical management approach. Treatment options include breast-conserving surgery, mastectomy, radiation therapy, and systemic therapy, with decisions currently most commonly driven by the more advanced and biologically aggressive site of the tumor. The prognosis of s-BBC is generally thought to be either somewhat worse or quite similar to that of unilateral breast cancer. Close surveillance with regular mammography and clinical follow-up is recommended for patients who undergo breast-conserving surgery to detect any new or recurrent breast cancer. Given the relative rarity of this clinical setting, further research is needed to determine the optimal management, risk assessment (i.e., appropriate application of prognostic molecular testing such as mammaprint or Oncotype), and risk-reduction strategies for patients with s-BBC.

## Figures and Tables

**Figure 3:**
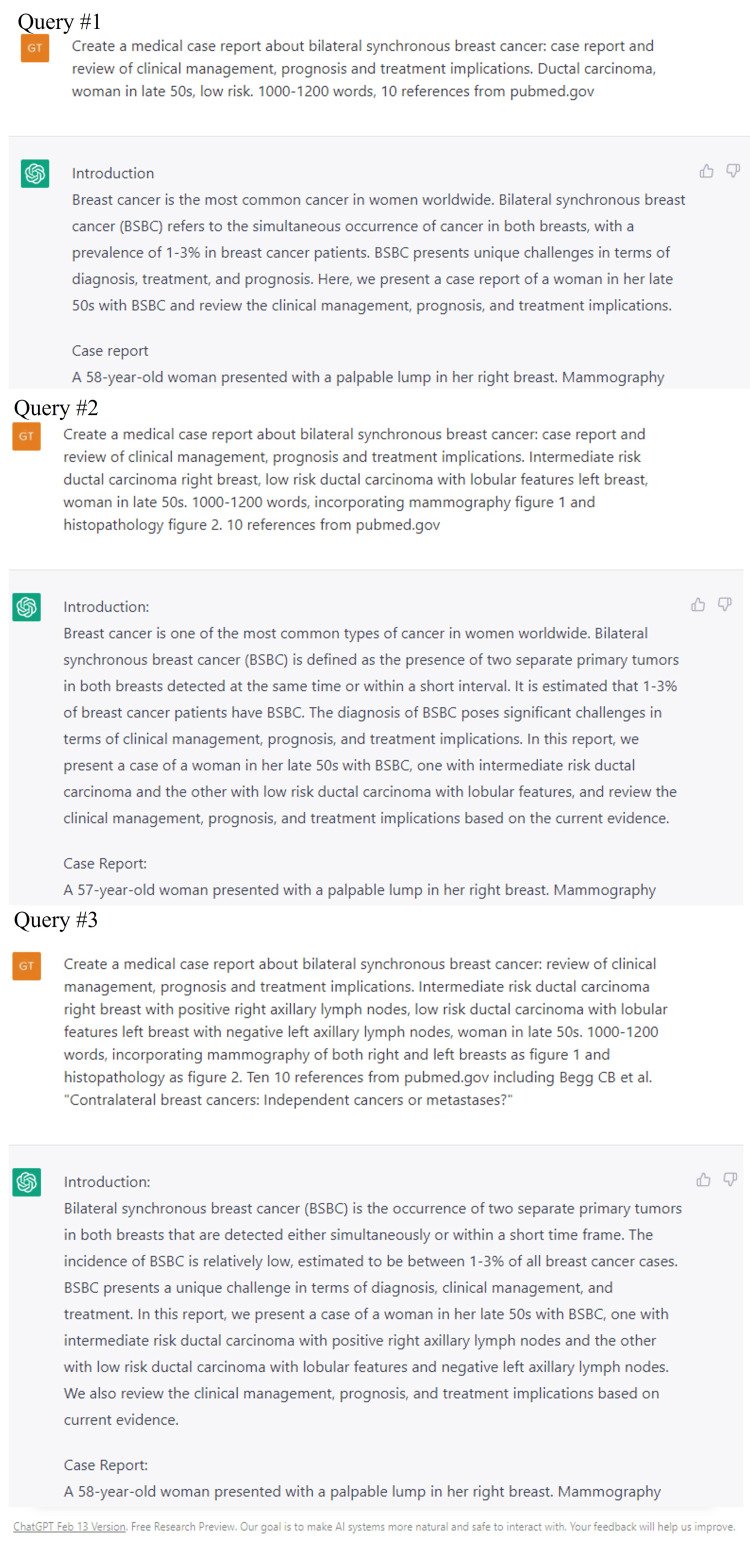
ChatGPT queries Three ChatGPT queries, stepwise more complex, with initial text generated. The final component of AI-generated text contained fragments from all three queries, but predominantly query #2.

**Figure 4:**
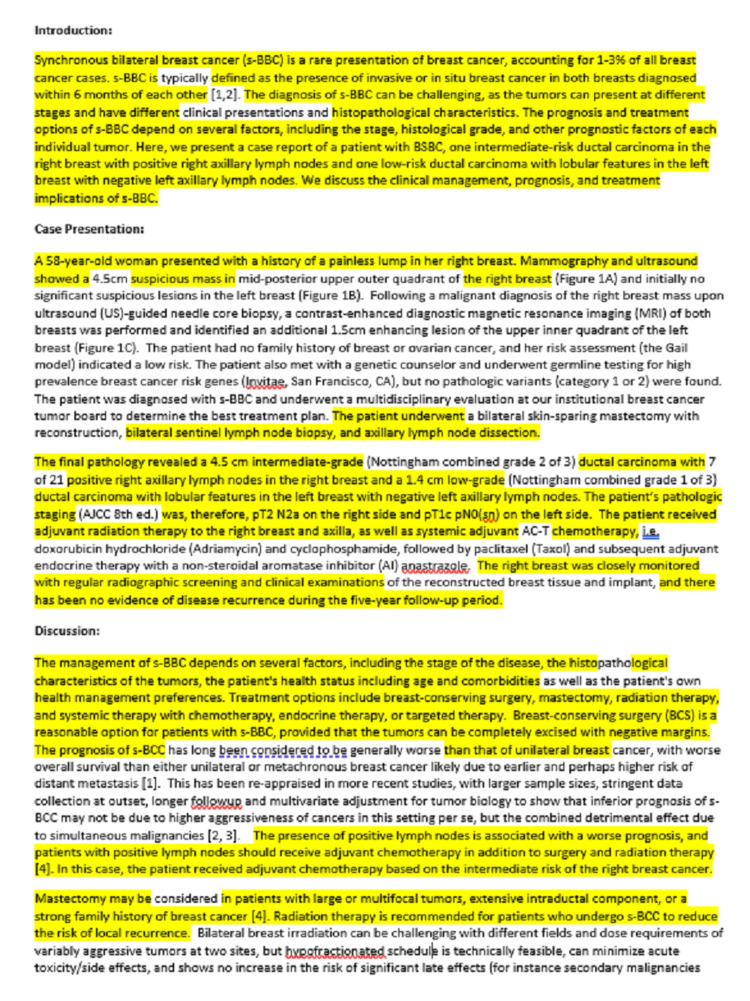
Script as generated by ChatGPT (Part A)

**Figure 5:**
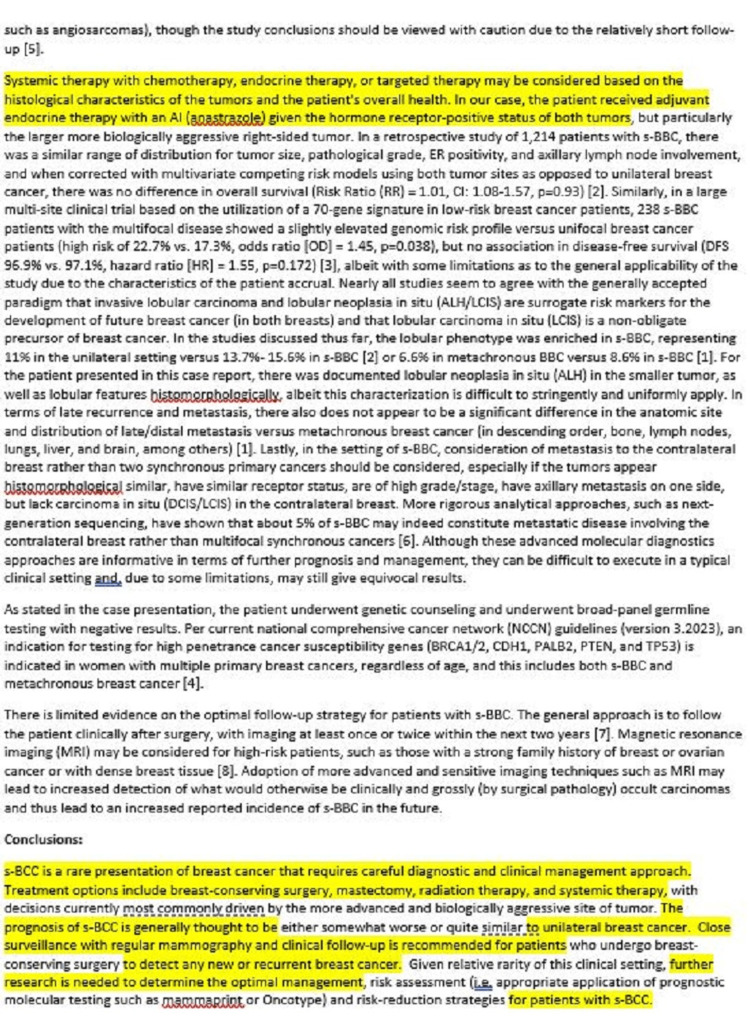
Script as Generated by ChatGPT (Part B)

## Appendices

Three similar queries were attempted with ChatGPT, and analyzed by GPTzero (https://gptzero.me/):

Ver 1:  Perplexity 39; Burstiness 15.7 (entirely AI)

Ver 2:  Perplexity 44; Burstiness 22 (partly AI)

Ver 3:  Perplexity 35; Burstiness 16.7 (entirely AI)

ChatGPT queries

One additional query (same as ver3) was attempted via BLOOM (a different LLM algorithm, trained with domain-specific text)

Perplexity 50.7; Burstiness 33.8 (partly AI)

For comparison, my most recent prior published case report is: Perplexity 120.3; Burstiness 88.8 (entirely human)

Lastly, the ChatGPT-assisted final version of the manuscript (~60% human text, discussion, and conclusion sections): Perplexity 62.3 Burstiness 45.8 (likely entirely human)

Methods: Default setting for GPTzero API (beta); at a threshold of 0.65, 85% of AI documents are classified as AI, and 99% of human documents are classified as human.

Author impressions (Dr. Gurda) 

Unfortunately, there is no such thing as a "free lunch" and not everything is as easy as it seems. The discussion sections generated by my queries to ChatGPT seemed to make sense, and superficially, the text generated appeared sensible, but the numbers and the citations produced were entirely fictitious. The authors and the journals existed; the article titles and numbers made general sense but were apparently pulled out of “thin air”, or rather generated by a confluence of text in the training set. As an example, ChatGPT text reads: 

"In a retrospective study of 166 patients with s-BBC who underwent BCS, the 10-year overall survival rate was 77%, and the disease-free survival rate was 67%.

The study found that the presence of lobular carcinoma in situ, an extensive intraductal component, and lymph node involvement were significant predictors of worse outcomes (1).

Sun J, Huo L, Xie C, et al. The prognosis of synchronous bilateral breast cancer: a single-center experience. PLoS One. 2015;10(6):e0128343."

It sounds credible, but this study does not exist. The authors exist, the journal exists, but the article and, most importantly, the data cited do not. Overall, for the purposes of generating a discussion section, ChatGPT at this point in its development (early 2023) appears to mostly have the characteristics of a 'confidence man' -- superficially accurate and strong, but the substance rings hollow.

Nonetheless, as stated elsewhere, I found ChatGPT and other LLMs to be useful in generating a skeletonized outline, organizing my thoughts about the subject in a logical manner (even if I had to gather the evidence for the arguments being made in the discussion 'manually' later), and in general as a motivation to get started and/or overcome writer's block.

Lastly, the full text of the article. What was generated by ChatGPT (verbatim) is highlighted, and manual human entry and edits are in standard text (Figures 4, 5):
